# Dorsal Vascular Complex Nonligation Method and Preservation of Puboprostatic Ligaments and Endopelvic Fascia During Laparoscopic Radical Prostatectomy: Effect on Continence

**DOI:** 10.5152/tud.2022.22113

**Published:** 2022-09-01

**Authors:** José Gaona, Margarita M. Zuluaga, Daniel H. Flórez, Francia M. Muñoz, Raul Rueda, Jairo Ortiz, Daniel E. Sánchez, Cesar Gonzalez, Fabio Gonzalez, Angélica M. Rueda, Sebastian Ortiz

**Affiliations:** 1 Universidad de Santander, Instituto Uromédica, Bucaramanga, Colombia

**Keywords:** Radical prostatectomy, prostate cancer, incontinence

## Abstract

**Objective:**

To evaluate the impact on continence rate during 1-year follow-up of a preservation technique that included nonligation of the dorsal vascular complex and sparing of the puboprostatic ligaments and the endopelvic fascia during laparoscopic radical prostatectomy.

**Material and methods:**

Information from 30 patients who underwent the preservation technique was prospectively collected and compared with data from 60 patients who underwent the nonpreservation traditional technique. A single surgeon performed all procedures.

**Results:**

Demographic and preoperative characteristics were similar. The mean patient age was 59 years in both groups. All patients were stage cT1c or cT2. Operative time was significantly lower in the preservation technique group (229.6 vs. 262.7 minutes, *P* < .001). There were no significant differences in intraoperative bleeding, discharge hemoglobin level, blood transfusion rate, length of hospitalization, and drop in the hemoglobin level. The probability of continence recovery was significantly higher in the preservation technique group than in the traditional technique group (hazard ratio = 0.50, 95% CI = 0.31-0.81). The continence rate (0 pads/day) for the preservation technique group versus the traditional technique group at 1, 3, 6, and 12 months was, respectively, 53.3% versus 30% (*P* = .031), 90% versus 45% (*P* < .001), 90% versus 63.3% (*P* = .008), and 96.6% versus 78.3% (*P* = .024). There were no significant differences between the groups regarding potency and oncologic outcomes.

**Conclusion:**

Nonligation of the dorsal vascular complex and preservation of the puboprostatic ligaments and the endopelvic fascia improved urinary continence compared with the traditional nonpreservation technique, with no impact in terms of bleeding and oncologic outcomes.

**Main Points**

- Dorsal vascular complex ligation, endopelvic fascia incision, and puboprostatic ligament incision are traditional steps during radical prostatectomy, potentially involved in the injury of the external sphincter complex.
- Nonligation of the dorsal vascular complex, nonincision of the endopelvic fascia, and nonincision of the puboprostatic ligaments during laparoscopic radical prostatectomy seem to impact the postoperative continence rate positively. The technique is not associated with a significant increase in the bleeding rate.
- The described preservation technique should be recommended and warrants further randomized comparative studies.

## Introduction

Postradical prostatectomy incontinence remains a significant issue, with an incidence of 4%-31%.^1-4^ Urinary incontinence has adverse effects on a patient’s quality of life because it produces shame, frustration, decreased cleanliness, and a diminished ability to enjoy daily activities.^5,6^ Postoperative leakage is mainly explained by an injury to any of the urethral sphincter complex components, such as the functional muscular mass, supporting structures, and coordinating neural pathways.^7,8^

Dorsal vascular complex (DVC) ligation, endopelvic fascia incision, and puboprostatic ligament incision are traditional steps during radical prostatectomy that might affect the integrity of the external sphincter complex. Dorsal vascular complex ligation carries the risk of entrapping the urethral sphincter’s functional muscular mass because the latter is covered laterally and dorsally by the DVC.^9,10^ The endopelvic fascia and puboprostatic ligaments are essential components of the anterior urethral support that stabilize the external sphincter and anchor the membranous urethra to the pubic bone.^11-13^ Additionally, preserving the endopelvic fascia prevents injury to the neural pathways of the urethral sphincter that are close to the levator ani muscle fascia.^7,14,15^

Therefore, we decided to perform a prospective evaluation of a preservation technique that included nonligation of the DVC, nonincision of the endopelvic fascia, and nonincision of the puboprostatic ligaments. In addition, patients who underwent the new procedure were compared to a cohort who underwent the traditional technique. Our primary aim was to determine the impact of the preservation technique on the postoperative continence rate. Secondary aims were to measure the effects on perioperative outcomes, complications, oncologic outcomes, and potency.

## Materials and Methods

**Figure 1.**
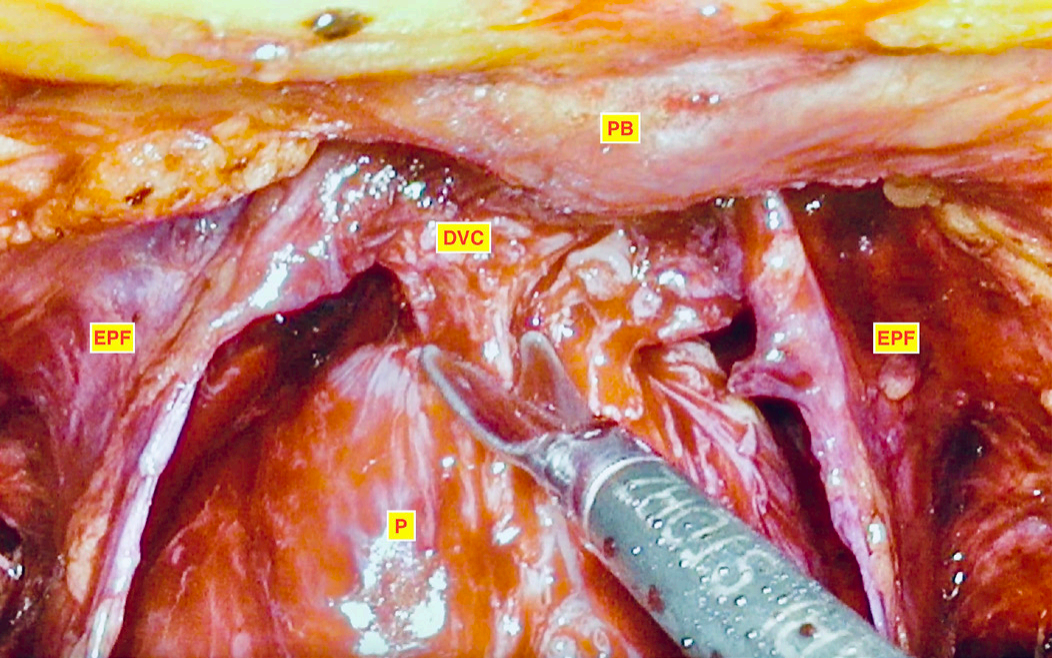
Preservation technique. The dorsal vascular complex (DVC) is transected with no previous ligation. There are no lateral incisions to the endopelvic fascia (EPF). P, prostate; PB, pubic bone.

We prospectively collected information from 30 consecutive patients who underwent radical prostatectomy with a 5-trocar laparoscopic preservation technique between November 2019 and August 2020. The surgical technique consisted of (1) transperitoneal trocar placement, (2) extended pelvic lymphadenectomy in cases with a lymph node metastasis probability ≥ 5% according to the Memorial Sloan Cancer Kettering Center nomogram,^16^ (3) dissection of the Retzius space, (4) transverse bladder neck incision, (5) athermal dissection of the vas deferens and seminal vesicles, (6) Denonvilliers’ fascia incision and posterior prostatic surface dissection, (7) prostate pedicle ligation, (8) bilateral posterolateral dissection, (9) apical dissection, (10) transection of the DVC and urethra (Figure 1), and (11) urethrovesical anastomosis with continuous 3-0 polydioxanone suture (Van Velthoven technique).

**Figure 2.**
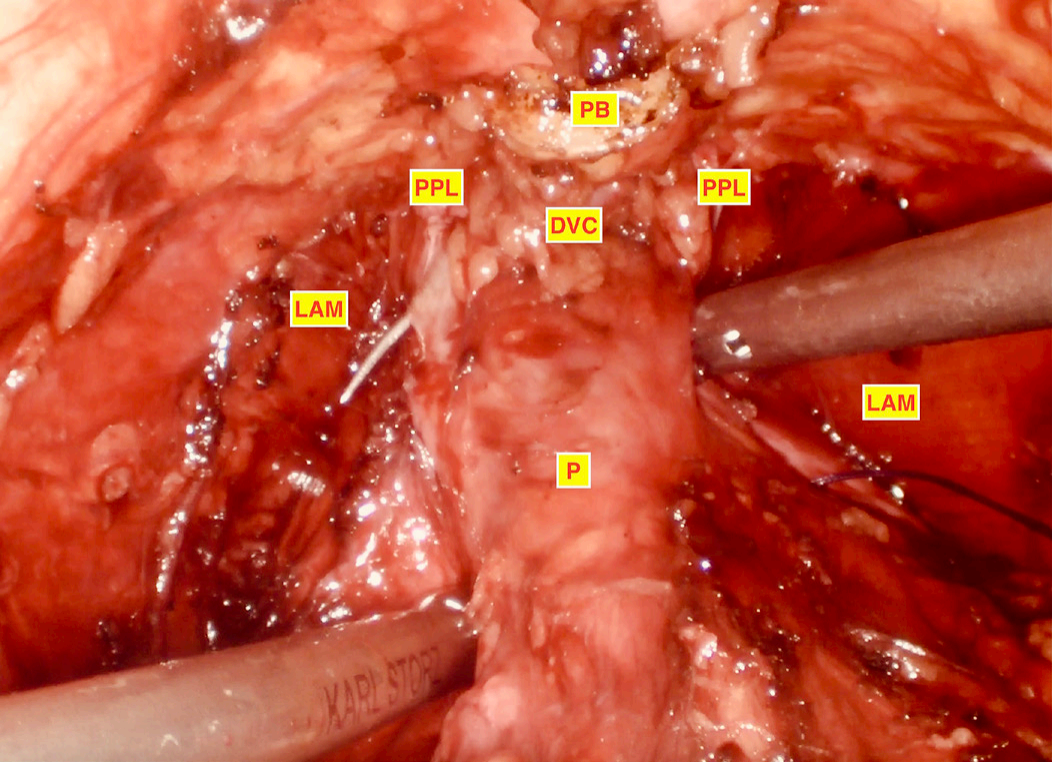
Traditional technique. The levator ani muscle (LAM) is exposed after a bilateral incision to the endopelvic fascia. The dorsal vascular complex (DVC) is ligated. The puboprostatic ligaments (PPL) are transected. P, prostate; PB, pubic bone.

In addition, we evaluated the medical records of 60 consecutive patients who underwent a traditional 5-trocar laparoscopic radical prostatectomy between December 2018 and October 2019. The traditional technique differed from the preservation technique in 3 steps, performed after the dissection of the Retzius space and before the transverse bladder neck incision (Figure 2): (1) bilateral endopelvic fascia incision, (2) bilateral puboprostatic ligament incision, and (3) DVC ligation with a single polyglactin 2-0 stitch.

The inclusion criteria were prostate cancer stage cT1 or cT2, a prostate-specific antigen (PSA) level <50 ng/mL, absence of bone metastases, and an age <70 years. Exclusion criteria were patients with neurogenic bladder or urinary incontinence before surgery. All procedures were performed by a single surgeon (J.G.) with over 400 cases of experience. Daily tadalafil (5 mg) was prescribed to all patients starting on the day of catheter removal until the recovery of sexual function. No bladder neck preservation or posterior urethral reconstruction was performed on any patient. The decision to perform side-specific incremental neurovascular preservation was done according to the nomogram developed by Martini et al.^17^ A 20-French urethral catheter remained in all patients for 10 days. Traction on the indwelling catheter was applied in the preservation technique group during the first 24 h. None of the patients received prophylaxis with low-molecular-weight heparin or pelvic floor therapy.

Preoperative, surgical, and postoperative variables are presented in Tables 1, 2, and 3, respectively. The PSA levels, continence rate, and potency rate were assessed at postoperative months 1, 3, 6, and 12. Continence was defined as the nonuse of pads. Patients were considered potent if they achieved sexual penetration with or without the use of phosphodiesterase-5 inhibitors. Prostate-specific antigen persistence was defined as a detectable postsurgical PSA level >0.2 ng/mL within 4 weeks after surgery. Biochemical recurrence was defined as 2 consecutive PSA values >0.2 ng/mL after an initial value of <0.2 ng/mL. The hemoglobin drop was the difference between preoperative and discharge hemoglobin levels. Our Institutional Ethics Committee approved this study (no.: 2018/07), and all patients provided informed consent.

### Statistical Analysis

The normal distribution of continuous variables was verified using the Kolmogorov–Smirnov test. Dichotomous variables are presented as absolute numbers and percentages. Continuous variables are presented as means and SD in the case of a normal distribution and medians and interquartile ranges in a non-normal distribution. The differences in proportions were determined using the chi-square test or Fisher’s exact test. Differences between groups with continuous variables were obtained using the Student’s *t*-test for variables with a normal distribution and the Mann–Whitney *U*-test for variables without a normal distribution. The survival graphs were prepared using the Kaplan–Meier method, and the difference between the survival curves was determined by the log-rank test. The size effect of the survival comparison was calculated using a simple Cox regression. A *P*-value <.05 was considered a statistically significant difference. The calculations were performed using Statistical Package for Social Sciences software version 17.0 (SPSS Inc.; Chicago, IL, USA). All *P*-values are 2-tailed.

## Results

Table 1 presents the demographic and preoperative characteristics of patients in the preservation (*n* = 30) and traditional (*n* = 60) technique groups. There were no significant differences in the age, body mass index (BMI), Charlson comorbidity index, PSA level, preoperative hemoglobin level, clinical stage, grade group, or D’Amico risk classification. The most common clinical stage was T2a (46.7%) in the preservation technique group and T1c (40%) in the traditional technique group. The most common specimen grade groups were 2 (30%) and 4 (30%) in the preservation technique group and 4 (31.7%) in the traditional technique group. Most patients in both groups were classified as intermediate or high risk.

Table 2 shows the perioperative outcomes. The operative time was significantly lower in the preservation technique group than in the traditional technique group (229.6 vs. 262.7 minutes, *P* < .001). There were no significant differences in the intraoperative bleeding, neurovascular bundle preservation rate, lymph node preservation rate, discharge hemoglobin level, blood transfusion rate, length of hospitalization, or drop in the hemoglobin level. There were significant differences in the number of complications between the groups. Clavien–Dindo grade I and grade II complications occurred in 1 (3.33%) and 6 (20%) patients in the preservation technique group and in 1 (1.66%) and 3 (5%) patients in the traditional technique group, respectively. There was 1 grade III complication in the preservation technique group (bladder clots) and 9 grade III complications in the traditional technique group (5 strictures of the fossa navicularis and 4 strictures of the vesicourethral anastomosis).

**Figure 3.**
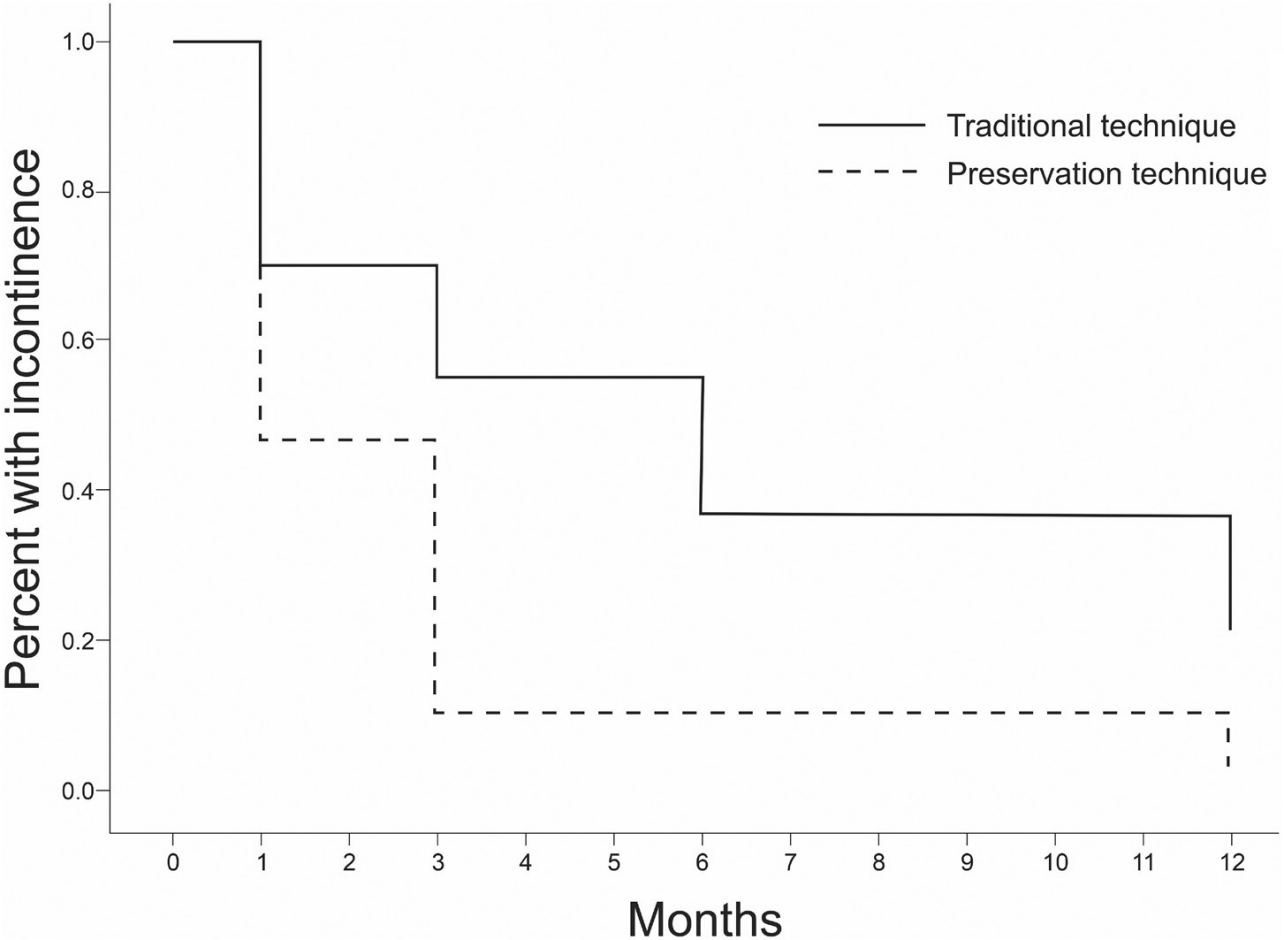
Kaplan–Meier curves of incontinence.

Table 3 shows the pathological, oncologic, and continence outcomes. There were no significant differences between the groups in terms of the prostate weight, specimen grade, pathological state, positive surgical margins (PSMs), PSA persistence, and biochemical recurrence. The most common specimen grade was 4 in both groups, accounting for 53.3% of the preservation technique group and 38.3% of the traditional technique group. Most cases were stage pT2, including 86.6% of the preservation technique group and 90% of the traditional technique group. The continence rate in the preservation technique group was significantly higher than that in the traditional technique group at 1 month (53.3% vs. 30%, respectively, *P* = .031), 3 months (90% vs. 45%, respectively, *P* < .001), 6 months (90% vs. 63.3%, respectively, *P* = .008), and 12 months (96.6% vs. 78.3%, respectively, *P* = .024). The Kaplan–Meier plot presents the continence rate over time (Figure 3). The median interval to achieve continence was 1 month in the preservation technique group and 6 months in the traditional technique group. The probability of continence recovery was significantly higher in the preservation technique group than in the traditional technique group [hazard ratio (HR) = 0.50, 95% CI = 0.31-0.81, *P* < .001)].

**Figure 4.**
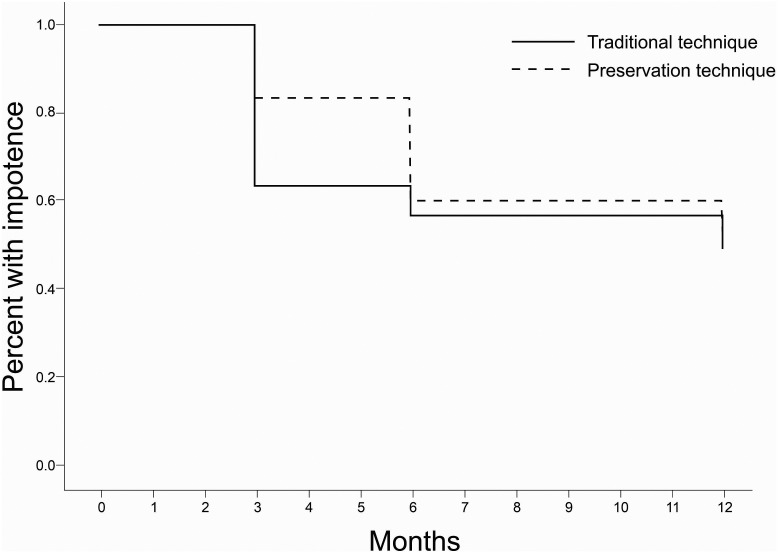
Kaplan–Meier curves of potence.

The median interval to achieve potency was not reached in the comparison groups*. *There was no significant difference between the groups in terms of the probability of potency recovery (HR = 1.24, 95% CI = 0.63-2.54, *P* = .43). Figure 4 shows the Kaplan–Meier potency rate curves over time.

## Discussion

This study evaluated the role of nonligation of the DVC, nonincision of the endopelvic fascia, and nonincision of the puboprostatic ligaments in the recovery of urinary continence after laparoscopic radical prostatectomy. Our procedure aimed to reduce the damage to the periprostatic structures involved in the continence mechanism. As a result, continence showed better recovery than the traditional nonpreservation technique.

The rationale for the nonligation of the DVC described in our study was based on anatomical and technical considerations. Ganzer et al^9^ demonstrated that the muscular mass of the urethral sphincter is at risk of injury during DVC ligation because at the prostate apex and 5 mm distal to it, 36.7% and 29.9% of the cross-sectional urethral sphincter surface area, respectively, are overlaid by the DVC. Thus, a DVC suture may include a significant ventral portion of the sphincter, especially if it is placed distal to the apex.^9,10^ Other anatomical studies have demonstrated the presence of neural somatic and autonomic branches that were lateral or anterior to the prostate apex and membranous urethra.^7,14^ These fibers are neural pathways for the cavernosal tissue and urethral sphincter and are at risk of damage if the DVC suture is too deep.^7,14^ On the other hand, the DVC ligature limits the mobility of the prostate apex and alters the apical anatomy, which implies a higher risk of positive margins due to an impaired circumferential visualization of the apex.^9,18,19^

Given the shortcomings of a DVC ligature, a selective and delayed DVC ligature has been proposed.^9,10,20^ The technique consists of transecting the DVC at the end of the prostatectomy, followed by selective suturing. Li et al^18^ published a meta-analysis of this technique, including 2 randomized trials and 6 retrospective studies. After 6 months of follow-up, there was a higher continence rate in the delayed ligature group [odds ratio (OR) = 1.46, 95% CI = 1.02-2.11, I2 = 3%, *P* = .04], but there were no differences in continence rates after 3 months (OR = 1.64, 95% CI = 0.98-2.73, I2 = 49%, *P* = .06) and 12 months (OR = 1.00, 95% CI = 0.63-1.57, I2 = 0%, *P* = .99). The low impact of the delayed ligature technique on the continence rate indicates that even selective stitches might injure the external sphincter muscular mass and supports our initiative to avoid stitching the DVC.

Few studies have reported a DVC nonligation method.^21-24^ Ferrara et al^22^ performed 150 intrafascial laparoscopic radical prostatectomies with transection but without DVC ligation and described a mean intraoperative bleeding amount of 220 mL and a transfusion rate of 3.3%. Cochetti et al^24^ described their experience with 210 patients who underwent a robotic extraperitoneal radical prostatectomy with anterograde intrafascial dissection and full DVC preservation, the Posterior, Extraperitoneal, Robotic, Under Santorini, Intrafascial, Anterograde (PERUSIA) technique. The median estimated blood loss was 150 mL, and the transfusion rate was 2.8%.^24^ Carvalho et al^23^ presented a 128-patient series involving robotic assisted DVC preservation and retrograde release of the neurovascular bundles, with a mean operative bleeding volume of 200 mL and a transfusion rate of 1.6%. Our mean transoperative bleeding (540 mL) and transfusion rates (10%) were higher than in previous studies. This can be explained by the DVC transection instead of complete preservation, as performed by Cochetti et al^24^ and Carvalho et al^23^ From our perspective, complete DVC preservation is difficult because of the medial DVC branches. Nevertheless, we did not observe a difference in the bleeding rate, transfusion rate, or drop in the hemoglobin level between the preservation and traditional technique groups. Therefore, our DVC nonligation method did not impact the bleeding.

The relatively low bleeding rate described in the literature with the DVC nonligation method relies on different technical aspects. First, the pneumoperitoneum CO_2_ pressure controls the venous bleeding, and the tiny arteries of the DVC can be easily controlled by pinpoint coagulation.^18,21,25^ Second, the DVC can be preserved, without transection, when an intrafascial prostatic apical dissection is performed underneath the DVC, as described by Carvalho et al^23^ and Cochetti et al.^[Bibr b23-tju-48-5-362] 24^ In this case, the DVC veins flow into the laterovesicoprostatic veins.^13^ Third, a metallic urethral sound can be inserted into the urethra after DVC transection, and pulling the distal tip of this device in an anterior direction compresses the DVC.^22^ Fourth, at the end of the surgery, traction of the indwelling urethral catheter can apply pressure over the bladder neck and occlude the DVC veins.^22^ We routinely used an 18-French metallic urethral sound after DVC transection and left 24-hour traction on the urethral catheter by placing an adhesive band between the catheter and thigh.

The preservation of the puboprostatic ligaments and the absence of an incision of the endopelvic fascia are additional components of our technique. The puboprostatic ligament is the most important anterior support structure of the urethral sphincter. Its contribution to continence by stabilizing the urethral complex has been previously described, including a prospective randomized study.^8,11,12,26,27^ The evidence regarding the benefit of endopelvic fascia preservation for postoperative continence is contradictory. While observational studies describe the benefit for the continence rate, a prospective randomized study found no difference in urinary continence between the groups.^15,28,29^ However, the rationale for preservation is firmly grounded in anatomical considerations. The endopelvic fascia is anterior support for the urethral sphincter. It is in proximity to nerves involved in continence and erectile function, including the pelvic plexus, neurovascular bundles, pudendal nerve branches to the rhabdosphincter, and somatic branches from pudendal or nonpudendal pathways that travel within the layers of the fascia of the levator ani.^7,14,15^ Therefore, if the dissection is too proximal or deep, any of these nerves may be affected.^7,10,14,15^ From our perspective, while endopelvic fascia preservation alone might not affect the postprostatectomy continence rate, it may play a role when accompanied by the preservation of other structures, such as the DVC and puboprostatic ligament.

Our comparative study found better continence with the preservation technique during the first-year of follow-up, with an HR of 0.5 (95% CI = 0.31-0.81) and continence rates (0 pads/day) of 53.34%, 90%, 90%, and 96.67% after 1, 3, 6, and 12 months of follow-up, respectively. Our results are similar to those of noncomparative studies that used a similar technique. Cochetti et al^24^ performed an anterograde robotic intrafascial preservation of the Veil of Aphrodite, the endopelvic fascia, the puboprostatic ligaments, and the DVC (the PERUSIA technique). The continence rates (0 pads/day) were 66.6%, 90.4%, and 96.1% after catheter removal, 3 months, and 12 months.^24^ Carvalho et al^23^ presented a robotic retrograde release of neurovascular bundles with preservation of the endopelvic fascia, puboprostatic ligaments, and DVC.^23^ They reported an immediate continence rate (0 pads/day) of 85.9% and continence rates of 94.5%, 97.7%, and 98.4% at 3, 6, and 12 months of follow-up, respectively.

Concerning the recovery of postoperative erectile function, we did not observe a significant difference between the periprostatic structure preservation and traditional technique groups (HR = 1.24, 95% CI = 0.63-2.54). After 12 months of follow-up, the median interval to achieve potency was not reached in the comparison groups. In contrast, the sexual potency rates reported by Carvalho et al^23^ and Cochetti et al^24^ at 12 months were 86.7% and 80.9%, respectively.^[Bibr b23-tju-48-5-362] [Bibr b24-tju-48-5-362]^ The impact of the DVC nonligation technique and endopelvic fascia and puboprostatic ligament preservation on the recovery of sexual function warrants further evaluation in prospective comparative studies.

Our study also compared the oncological and perioperative outcomes between the preservation and traditional technique groups. The preservation technique demonstrated adequate oncologic results, with PSMs and biochemical recurrence rates similar to those of the traditional technique group. The PSM and biochemical recurrence rates in the preservation technique were 13.6% and 6.6%, respectively, similar to prior reports.^30^ Omitting steps, such as ligation of the DVC and periprostatic structure incisions, led to a shorter surgical time with the preservative technique (229.6 vs. 262.7 minutes, *P* < .01). The complication rate was similar between the groups in terms of intraoperative bleeding, transfusion rates, and drop in hemoglobin levels. The results demonstrate the effectiveness of our strategies for DVC control after transection, including the use of a metallic urethral sound and traction on the urethral catheter, as discussed earlier.

Our study has several strengths. To our knowledge, this is the first study to compare DVC ligation with a nonligation method. The comparison groups were similar in terms of patient characteristics and tumor risk, thereby reducing selection bias. Data from the preservation group technique were collected prospectively. The distribution of variables that might affect the continence rate (age, BMI, prostate volume, Charlson comorbidity index, and neurovascular bundle preservation) was similar in both groups.^2,26^ Finally, the procedures were performed by a single surgeon, thus limiting the variation associated with the surgeon’s technique.

The current study has some limitations that should be mentioned. First, the analysis was based on a relatively small sample size. Second, we did not include preexisting lower urinary tract symptoms and the magnetic resonance estimation of the urethral length, which are predictors of postprostatectomy incontinence.^12,26^ Third, the use of a single laparoscopic surgeon affects the generalizability of the results. Fourth, the quality of the presented potency outcomes is affected by the lack of measurements based on validated questionnaires, such as the International Index of Erectile Function-5 score. Fifth, data from the traditional technique group were prone to information bias because they were collected retrospectively. Finally, the period between the 2 groups differed, affecting the results because of different surgeons’ experience levels.

In conclusion, our study demonstrated that the DVC nonligation technique, accompanied by sparing the endopelvic fascia and puboprostatic ligaments, led to a significant improvement in the continence rate during the first-year follow-up after laparoscopic radical prostatectomy compared to the traditional method. The technique did not increase the intraoperative and postoperative bleeding rates and did not affect oncologic outcomes. Based on our results, our preservation technique might be recommended over the standard surgical method and warrants further randomized comparative analyses.

## Figures and Tables

**Table 1.** Demographic and Preoperative Features

Variable	Preservation Technique (n = 30)	Traditional Technique (n = 60)	*P*
Age (years), median ± SD	59.73 ± 6.61	59.6 ± 6.25	.92
BMI, mean ± SD	25.28 ± 2.86	26.96 ± 3.66	.05
Charlson comorbidity index, median (IQR)	3 (3-4)	4 (3-4)	.56
PSA, median (IQR)	7.85 (6.22-10.07)	8 (6.15-12)	.58
Preoperative hemoglobin, mean ± SD	14.83 ± 1.18	15.25 ± 1.32	.36
Clinical stage, n (%)			
T1c	9 (30)	24 (40)	.1
T2a	14 (46.7)	15 (25)	
T2b	7 (34.3)	16 (26.7)	
T2c	0 (0)	5 (8.3)
Specimen grade group, *N* (%)			
1	6 (20)	15 (25)	.87
2	9 (30)	12 (20)	
3	4 (13.3)	10 (16.7)	
4	9 (30)	19 (31.7)	
5	2 (6.7)	4 (6.7)
D’Amico risk classification, *N* (%)			
Low risk	4 (13.33)	8 (13.3)	.98
Intermediate risk	13 (43.33)	25 (41.66)	
High risk	13 (43.33)	27 (45.01)

BMI, body mass index; IQR, interquartile range; PSA, prostate-specific antigen.

**Table 2.** Perioperative Outcomes

Variable	Preservation Technique (n = 30)	Traditional Technique (n = 60)	*P*
Operative time (minutes), mean ± SD	229.67 ± 47.16	262.75 ± 38.43	<.001
Intraoperative bleeding (mL), mean ± SD	540 ± 365.73	574.35 ± 334.93	.72
Neurovascular bundle preservation, *N* (%)			
Unilateral	5 (16.7)	6 (10)	.05
Bilateral	25 (83.3)	44 (73.3)	
None	0 (0)	10 (16.7)
Lymph node dissection, *N* (%)	18 (60)	28 (46.7)	.26
Discharge hemoglobin level, mean ± SD	10.85 ± 1.57	11.01 ± 1.78	.79
Blood transfusion, *N* (%)	3 (10)	3 (5)	.39
Length of hospitalization (hours), median (IQR)	75 (71.5-100)	91.5 (72-119.76)	.31
Drop in hemoglobin level, mean ± SD	3.98 ± 1.97	4.23 ± 1.65	.69
Clavien–Dindo complications, *N* (%)			
Grade I	1 (3.33)	1 (1.66)	.024
Grade II	6 (20)	3 (5)	
Grade III	1 (3.33)	9 (15)

IQR, interquartile range.

**Table 3.** Pathologic, Oncologic, and Continence Outcomes

Variable	Preservation Technique (n = 30)	Traditional Technique (n = 60)	*P*
Pathological prostate weight (g), mean ± SD	40.86 ± 14.15	41.91 ± 14.43	.75
Pathological specimen grade group, *N* (%)			
1	2 (6.7)	9 (15)	.43
2	5 (16.7)	12 (20)	
3	7 (23.3)	13 (21.7)	
4	16 (53.3)	23 (38.3)	
5	0 (0)	3 (5)
Pathological stage, *N* (%)			
pT2	26 (86.6)	54 (90)	.72
pT3	4 (13.3)	6 (10)
Positive surgical margins, *N* (%)	4 (13.3)	10 (16.6)	.76
PSA persistence, *N* (%)	1 (3.3)	2 (3.3)	1
Biochemical recurrence, *N* (%)	2 (6.6)	10 (16.6)	.32
Continence rate, *N* (%)			
1 month	16 (53.3)	18 (30)	.031
3 month	27 (90)	27 (45)	<.001
6 month	27 (90)	38 (63.3)	.008
12 month	29 (96.6)	47 (78.3)	.024

PSA, prostate-specific antigen.

## References

[1] Urkmez A Ranasinghe W Davis JW . Surgical techniques to improve continence recovery after robot-assisted radical prostatectomy. Transl Androl Urol. 2020;9(6):3036 3048. 10.21037/tau.2020.03.36) 33457277 PMC7807332

[2] Ficarra V Novara G Rosen RC et al. Systematic review and meta-analysis of studies reporting urinary continence recovery after robot-assisted radical prostatectomy. Eur Urol. 2012;62(3):405 417. 10.1016/j.eururo.2012.05.045) 22749852

[3] Collette ERP Klaver SO Lissenberg-Witte BI van den Ouden D van Moorselaar RJA Vis AN . Patient-reported outcome measures concerning urinary incontinence after robot assisted radical prostatectomy: development and validation of an online prediction model using clinical parameters, lower urinary tract symptoms and surgical experience. J Robot Surg. 2021;15(4):593 602. 10.1007/s11701-020-01145-9) 32930971 PMC8295126

[4] Hagman A Lantz A Carlsson S et al. Urinary continence recovery and oncological outcomes after surgery for prostate cancer analyzed by risk category: results from the laparoscopic prostatectomy robot and open trial. World J Urol. 2021;39(3):3239 3249 33743059 10.1007/s00345-021-03662-0

[5] García Cortés Á Colombás Vives J Gutiérrez Castañé C et al. What is the impact of post-radical prostatectomy urinary incontinence on everyday quality of life? Linking Pad usage and International Consultation on Incontinence Questionnaire Short-Form (ICIQ-SF) for a COMBined definition (PICOMB definition). Neurourol Urodyn. 2021;40(3):840 847. 10.1002/nau.24631) 33604977

[6] Clark JA Inui TS Silliman RA et al. Patients’ perceptions of quality of life after treatment for early prostate cancer. J Clin Oncol. 2003;21(20):3777 3784. 10.1200/JCO.2003.02.115) 14551296

[7] Bessede T Sooriakumaran P Takenaka A Tewari A . Neural supply of the male urethral sphincter : comprehensive anatomical review and implications for continence recovery after radical prostatectomy. World J Urol. 2017;35(4):549 565. 10.1007/s00345-016-1901-8) 27484205

[8] Tan GY El Douaihy Y Te AE Tewari AK . Scientific and technical advances in continence recovery following radical prostatectomy. Expert Rev Med Devices. 2009;6(4):431 453. 10.1586/erd.09.19) 19572798

[9] Ganzer R Stolzenburg JU Neuhaus J Weber F Burger M Bründl J . Is the striated urethral sphincter at risk by standard suture ligation of the dorsal vascular complex in radical prostatectomy? An anatomic study. Urology. 2014;84(6):1453 1458. 10.1016/j.urology.2014.06.092) 25432837

[10] Walz J Epstein JI Ganzer R et al. A critical analysis of the current knowledge of surgical anatomy of the prostate related to optimisation of cancer control and preservation of continence and erection in candidates for radical prostatectomy: an update. Eur Urol. 2016;70(2):301 311. 10.1016/j.eururo.2016.01.026) 26850969

[11] Kojima Y Takahashi N Haga N et al. Urinary incontinence after robot-assisted radical prostatectomy: pathophysiology and intraoperative techniques to improve surgical outcome. Int J Urol. 2013;20(11):1052 1063. 10.1111/iju.12214) 23841851

[12] Heesakkers J Farag F Bauer RM Sandhu J De Ridder D Stenzl A . Pathophysiology and contributing factors in postprostatectomy incontinence: a review. Eur Urol. 2017;71(6):936 944. 10.1016/j.eururo.2016.09.031) 27720536

[13] Herranz Amo F Radical retropubic prostatectomy: control of Santorini’s venous plexus. Actas Urol Esp (Engl). 2020;44(6):417 422. 10.1016/j.acuro.2020.04.009 32507362

[14] Tewari A Takenaka A Mtui E et al. The proximal neurovascular plate and the tri-zonal neural architecture around the prostate gland: importance in the athermal robotic technique of nerve-sparing prostatectomy. BJU Int. 2006;98(2):314 323. 10.1111/j.1464-410X.2006.06266.x) 16879671

[15] Takenaka A Hara R Soga H Murakami G Fujisawa M . A novel technique for approaching the endopelvic fascia in retropubic radical prostatectomy, based on an anatomical study of fixed and fresh cadavers. BJU Int. 2005;95(6):766 771. 10.1111/j.1464-410X.2005.05397.x) 15794779

[16] Hinev AI Anakievski D Kolev N Marianovski V Hadjiev V . Validation of pre- and postoperative nomograms used to predict the pathological stage and prostate cancer recurrence after radical prostatectomy: a multi-institutional study. J BUON. 2011;16(2):316 322. 21766504

[17] Martini A Gupta A Lewis SC et al. Development and internal validation of a side-specific, multiparametric magnetic resonance imaging-based nomogram for the prediction of extracapsular extension of prostate cancer. BJU Int. 2018;122(6):1025 1033. 10.1111/bju.14353) 29676063

[18] Li H Chen J Cui Y Liu P Yi Z Zu X . Delayed versus standard ligature of the dorsal venous complex during laparoscopic radical prostatectomy: a systematic review and meta-analysis of comparative studies. Int J Surg. 2019;68(June):117 125. 10.1016/j.ijsu.2019.06.015) 31271930

[19] Pan JW Jin XW Luo FX et al. Beforehand transection and suturing (Bts) of the dorsal vascular complex: a novel technique in laparoscopic radical prostatectomy. Gland Surg. 2020;9(6):2116 2124. 10.21037/gs-20-813) 33447562 PMC7804557

[20] Porpiglia F Fiori C Grande S Morra I Scarpa RM . Selective versus Standard Ligature of the Deep Venous Complex during laparoscopic radical prostatectomy: effects on Continence, Blood Loss, and Margin Status. Eur Urol. 2009;55(6):1377 1383. 10.1016/j.eururo.2009.02.009) 19243886

[21] Power NE Silberstein JL Kulkarni GS Laudone VP . The dorsal venous complex (DVC): dorsal venous or dorsal vasculature complex? Santorini’s plexus revisited. BJU Int. 2011;108(6):930 932. 10.1111/j.1464-410X.2011.10586.x) 21884359 PMC4315333

[22] Ferrara V Giannubilo W Azizi B Vecchioli Scaldazza C Garritano A . Prostatectomia radicale video-laparoscopica senza legatura del plesso venoso del Santorini [Laparoscopic radical prostatectomy without ligation of the Santorini`s venous plexus]. Urologia. 2010;77(1):57 62. Italian. 10.1177/039156031007700110) 20890860

[b23-tju-48-5-362] de Carvalho PA Barbosa JABA Guglielmetti GB et al. Retrograde release of the neurovascular bundle with preservation of dorsal venous complex During robot-assisted radical prostatectomy: optimizing functional outcomes. Eur Urol. 2020;77(5):628 635. 10.1016/j.eururo.2018.07.003) 30041833

[b24-tju-48-5-362] Cochetti G Boni A Barillaro F Pohja S Cirocchi R Mearini E . Full neurovascular sparing extraperitoneal robotic radical prostatectomy: our experience with PERUSIA technique. J Endourol. 2017;31(1):32 37. 10.1089/end.2016.0477) 27824258

[25] Sasaki H Miki J Kimura T et al. Upfront transection and subsequent ligation of the dorsal vein complex during laparoscopic radical prostatectomy. Int J Urol. 2010;17(11):960 961. 10.1111/j.1442-2042.2010.02632.x) 20868442

[26] Schifano N Capogrosso P Tutolo M Dehò F Montorsi F Salonia A . et al. World J Mens Health. 2021;39(4):581 597. 10.5534/wjmh.200114) 33151045 PMC8443978

[27] Assem A Youssif TA Hamdy SM Beltagy AM . Role of sparing of puboprostatic ligaments on continence recovery after radical prostatectomy: a randomized controlled trial. Scand J Urol. 2020:1–5. 10.1080/21681805.2020.1849389 33241757

[28] Kwon SY Lee JN Kim HT et al. Endopelvic fascia preservation during robot-assisted laparoscopic radical prostatectomy: does it affect urinary incontinence? Scand J Urol. 2014;48(6):506 512. 10.3109/21681805.2014.913259) 25008957

[29] Siltari A Riikonen J Murtola TJ . Preservation of endopelvic fascia: effects on postoperative incontinence and sexual function - A randomized clinical trial. J Sex Med. 2021;18(2):327 338. 10.1016/j.jsxm.2020.11.003) 33358241

[30] Huang X Wang L Zheng X Wang X . Comparison of perioperative, functional, and oncologic outcomes between standard laparoscopic and robotic-assisted radical prostatectomy: a systemic review and meta-analysis. Surg Endosc. 2017;31(3):1045 1060. 10.1007/s00464-016-5125-1) 27444830

